# Longitudinal Changes of White Matter Hyperintensities in Sporadic Small Vessel Disease: A Systematic Review and Meta-analysis

**DOI:** 10.1212/WNL.0000000000201205

**Published:** 2022-11-29

**Authors:** Angela C.C. Jochems, Carmen Arteaga, Francesca Chappell, Tuula Ritakari, Monique Hooley, Fergus Doubal, Susana Muñoz Maniega, Joanna M. Wardlaw

**Affiliations:** From the Centre for Clinical Brain Sciences (A.C.C.J., C.A., F.C., T.R., F.D., S.M.M., J.M.W.), UK Dementia Research Institute (A.C.C.J., C.A., F.C., T.R., F.D., S.M.M., J.M.W.), and Centre for Discovery Brain Sciences (M.H.), University of Edinburgh, United Kingdom.

## Abstract

**Background and Objectives:**

White matter hyperintensities (WMHs) are frequent imaging features of small vessel disease (SVD) and related to poor clinical outcomes. WMH progression over time is well described, but regression was also noted recently, although the frequency and associated factors are unknown. This systematic review and meta-analysis aims to assess longitudinal intraindividual WMH volume changes in sporadic SVD.

**Methods:**

We searched EMBASE and MEDLINE for articles up to 28 January 2022 on WMH volume changes using MRI on ≥2 time points in adults with sporadic SVD. We classified populations (healthy/community-dwelling, stroke, cognitive, other vascular risk factors, and depression) based on study characteristics. We performed random-effects meta-analyses with Knapp-Hartung adjustment to determine mean WMH volume change (change in milliliters, percentage of intracranial volume [%ICV], or milliliters per year), 95% CI, and prediction intervals (PIs, limits of increase and decrease) using unadjusted data. Risk of bias assessment tool for nonrandomized studies was used to assess risk of bias. We followed Preferred Reporting in Systematic Review and Meta-Analysis guidelines.

**Results:**

Forty-one articles, 12,284 participants, met the inclusion criteria. Thirteen articles had low risk of bias across all domains. Mean WMH volume increased over time by 1.74 mL (95% CI 1.23–2.26; PI −1.24 to 4.73 mL; 27 articles, N = 7,411, mean time interval 2.7 years, SD = 1.65); 0.25 %ICV (95% CI 0.14–0.36; PI −0.06 to 0.56; 6 articles, N = 1,071, mean time interval 3.5 years, SD = 1.54); or 0.58 mL/y (95% CI 0.35–0.81; PI −0.26 to 1.41; 8 articles, N = 3,802). In addition, 13 articles specifically mentioned and/or provided data on WMH regression, which occurred in asymptomatic, stroke, and cognitive disorders related to SVD.

**Discussion:**

Net mean WMH volume increases over time mask wide-ranging change (e.g., mean increase of 1.75 mL ranging from 1.25 mL decrease to 4.75 mL increase), with regression documented explicitly in up to one-third of participants. More knowledge on underlying mechanisms, associated factors, and clinical correlates is needed, as WMH regression could be an important intervention target.

White matter hyperintensities (WMHs) of presumed vascular origin are the most common neuroimaging feature of small vessel disease (SVD), a disorder of the cerebral microvessels. WMHs are visible on MRI as hyperintense lesions on T2-weighted and hypointense on T1-weighted sequences.^1^

Around 11%–15% of general middle-aged population have 1 or more WMHs, increasing to over 90% in people older than 80 years. WMHs are associated with risk factors including age, hypertension, smoking, and diabetes,^2^ higher WMH volume at baseline,^2^ and with symptoms such as apathy, fatigue, delirium, cognitive decline,^3^ and increased risk of falls,^4^ stroke and dementia.^3^

WMHs are thought to indicate areas of permanent white matter damage due to demyelination and axon loss.^3^ In longitudinal studies, WMH progression over time is a common finding, whereas WMH regression has only been noted in a few recent studies,^5,6^ but otherwise has been disregarded as measurement error or overlooked. If a genuine finding, then WMH regression might suggest that WMHs do not only indicate permanently damaged brain tissue. A better understanding of the frequency and factors associated with WMH regression could help identify potential interventions to delay WMH progression and the devastating clinical consequences.

We hypothesize that WMH regression might be a wider phenomenon than what has been reported so far. Therefore, our aim was to assess longitudinal intraindividual WMH volume changes in sporadic SVD over time using volumetric MRI measurements and progression rates in this systematic review and meta-analysis.

## Methods

### Standard Protocol Approvals, Registrations, and Patient Consents

We registered the protocol on PROSPERO, an international prospective register for systematic reviews, on January 23, 2018 (Registration Number CRD42018080548). The reporting of the systematic review and meta-analysis follows the Preferred Reporting in Systematic Review and Meta-Analysis guidelines.

### Search Strategy and Selection Criteria

We searched EMBASE and MEDLINE from January 1985, when MRI became more widely implemented in clinical practice, to January 28, 2022, for studies investigating longitudinal quantification of WMH volume on MRI on at least 2 different times points in adults older than 18 years (eAppendix 1: search strategy, links.lww.com/WNL/C292). We supplemented this search with hand-searched articles from January 2012 to December 22, 2020, in *Stroke*, *Journal of Cerebral Blood Flow and Metabolism*, and *Neurology*®. These journals were chosen because they are outstanding peer-reviewed journals that publish articles on community-dwelling participants and patients with sporadic SVD. Contents of the journals were screened for relevant articles based on title and abstract. The screening process is described below.

We included published full-text articles from peer-reviewed longitudinal studies that used MRI to quantify WMH volumes, defined according to STandards for ReportIng Vascular changes on nEuroimaging criteria,^1^ on at least 2 different time points and that provided numerical analysis of WMH volume change between the time points. Studies included randomized trials, nonrandomized trials, cohort studies, and case-control studies. For data from a single cohort published more than once, we included the most relevant article with the largest sample size and years of follow-up and most useable data to minimize duplication or overlapping samples. We excluded studies of SVD attributable to hereditary causes (e.g., cerebral autosomal dominant arteriopathy with subcortical infarcts and leukoencephalopathy and cerebral autosomal recessive arteriopathy with subcortical infarcts and leukoencephalopathy) or WMH attributable to other causes (e.g., multiple sclerosis, inflammatory disorders including primary angiitis, secondary vasculitis, postinfectious, and paraneoplastic syndromes).

Title and abstract screening and duplicate article removal were performed independently by 1 reviewer (C.A., A.C.C.J., T.R., or M.H.). A second reviewer screened a random 10% sample of titles and abstracts. Full-text review was assessed independently by 1 reviewer, and a second reviewer screened a random 20% sample of the full texts (C.A. or A.C.C.J.) using Covidence software. Data extraction was performed by a single reviewer using a prespecified data collection form (eTable 1, links.lww.com/WNL/C292), and a second reviewer double extracted a random 20% sample. Two reviewers used risk of bias assessment tool for nonrandomized studies^7^ to assess all studies including randomized trials, for participant selection, adjustment for confounders, adequacy of WMH descriptors, blinding, incomplete outcome data, and selective outcome reporting (eTable 2). Any disagreements were resolved by discussion between reviewers (C.A. and A.C.C.J.) with the help of a senior reviewer (J.M.W.).

### Data Extraction

We extracted information on study design, demographic characteristics, vascular risk factors (VRFs), study population (i.e., healthy and/or community-dwelling population, patients with depression, and patients with stroke, cognitive disorders, or other VRF presentations), and follow-up period. We extracted data related to MRI assessment, including WMH volumes per time point and WMH volume changes between those time points, any adjustments of WMH measurements to, for example, intracranial volume or total brain volume; methods of WMH calculation and predictors of change. WMH volume data are often skewed and are log transformed to normalize the data before being used in analyses. We aimed to use unadjusted raw data where possible to reflect real intraindividual changes and include the whole range of least and most growth. We selected studies that provided unadjusted mean raw WMH volume change, for example, mL, cm^3^ and cc, changes in volume expressed as a percentage of intracranial volume (%ICV), or an annual change rate (e.g., milliliters per year). We extracted the mean or median with corresponding SD, interquartile range (IQR), range, 95% CI, SEM, and, if provided, the baseline WMH volumes. If studies did not provide mean and SD of WMH volume change, we calculated the mean and SD from the sample size, median, range, IQR, SEM, or 95% CI.^8^ We extracted data from studies that reported volumes per group and for the entire cohort; when the volume for the entire cohort was not available, data for the separate groups were included. For studies that did not report complete volumetric assessments, or where the data were not useable in the meta-analysis, we extracted relevant measures to perform a narrative summary of findings.

### Statistical Analysis

We used random-effects meta-analyses (because of expected between-study heterogeneity) to calculate mean WMH change, its CI, and prediction interval (PI) using untransformed raw means. More than 2 studies are needed to calculate a PI. The PI is an estimate of an interval, based on data that have already been observed and are included in the meta-analysis, in which future observations will fall, with 95% CI. In contrast to the CI, it indicates the range of least and most WMH volume change in a sample, whereas the CI indicates the range of mean change. We applied the Knapp-Hartung adjustment to control for uncertainty regarding between-study heterogeneity and calculate the CI around the pooled mean.^9^ We planned additional explorative subgroup analyses by study population group (e.g., stroke), age strata, and time lapse between MRIs, when possible. Populations were assigned based on the study characteristics. When articles reported subgroups, for example, cases, controls, or treatment groups, these were entered as separate groups and mentioned in the main meta-analyses unless data from the total group were used. However, there were insufficient data to assess trial interventions. We assessed heterogeneity by visual assessment of the forest plots and by calculating the *I*^2^ and *τ*^2^ statistics to estimate the between-study variance with a restricted maximum-likelihood estimator. We used R version 4.0.2 and the meta package.^10^

### Data Availability

Data used in this study are available on reasonable request from the corresponding author.

## Results

**Figure 1:**
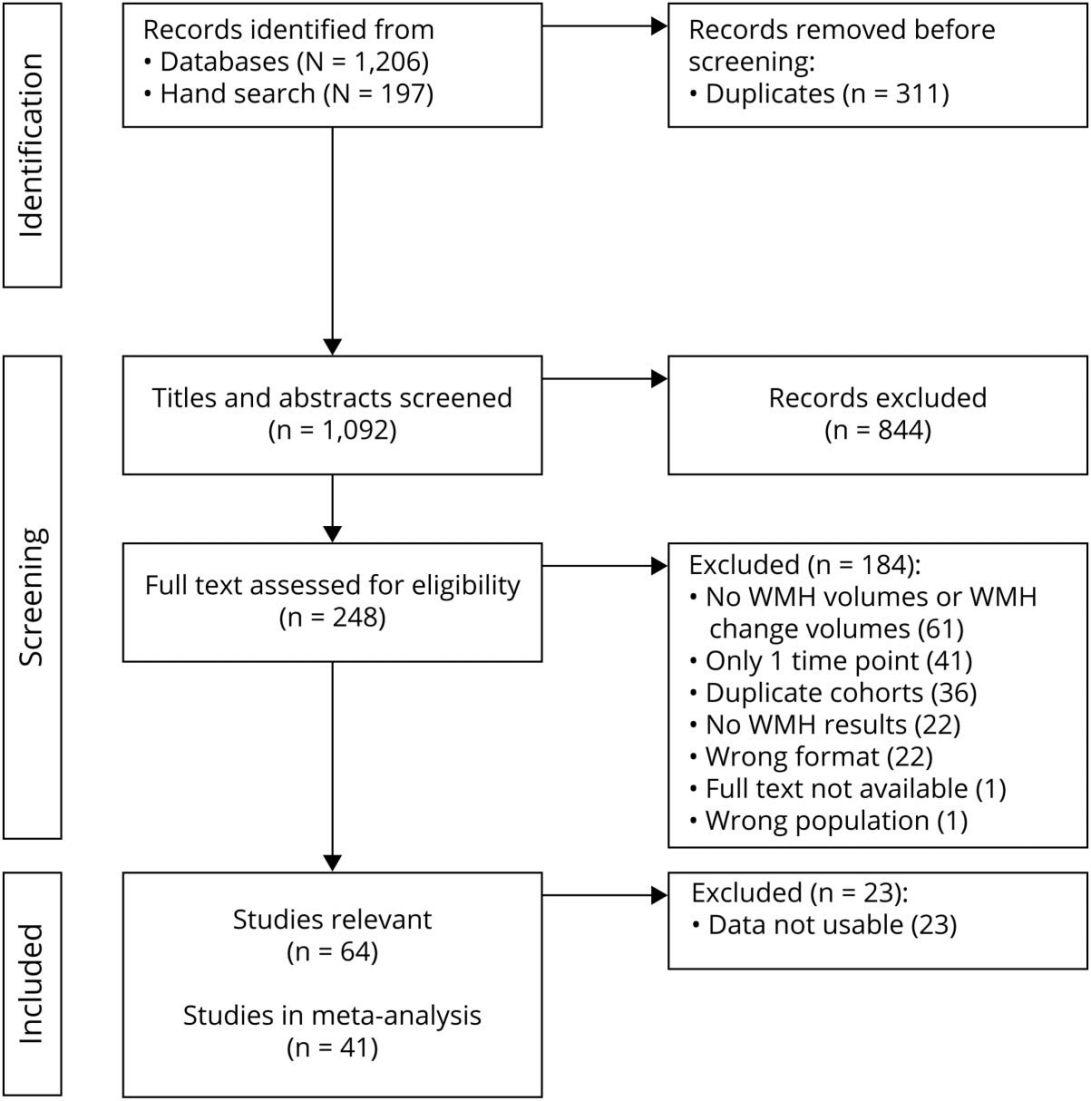
Flowchart of Study Selection WMH = white matter hyperintensity.

Our search yielded 1,206 publications, and our manual search provided 197 further publications (Figure 1). After title and abstract screening, 248 full texts were assessed for eligibility. Most articles were excluded because they only reported WMH volumes at 1 time point, reported no WMH volumes at all, or were superceded by a publication from the same study reporting more complete relevant data. This left 64 articles that were relevant to the review question, but 23 articles did not provide raw/unadjusted WMH volumes and instead used log-transformed, estimated volumes or percentages of change compared with baseline volumes. Therefore, 41 articles were included in the final meta-analyses. These 41 articles comprised 39 different studies, 12,284 participants (summarized in eTables 3–5, links.lww.com/WNL/C292), and had a median time between scans of 2.6 years (range 0.25-8.7 years). For 13/41 articles included in the meta-analysis, we had to estimate the mean and SD. The 23 relevant articles that did not provide useable WMH change data are summarized in text, eAppendix 2 and eTable 6.

### Risk of Bias

Thirteen articles had a low risk of bias across all the domains. Four articles had high risk of bias in 1 domain, 2/4 the bias was incomplete outcome data and for 2/4 there was high risk for inadequate consideration of confounding variables. The remaining 24 articles had 1 or 2 domains where the risk was unclear according to the reviewing authors; the main biases here were blinding of outcome assessments and incomplete outcome data (eTable 2, links.lww.com/WNL/C292).

### Intraindividual WMH Change

#### WMH Volume Change in Milliliters

**Figure 2:**
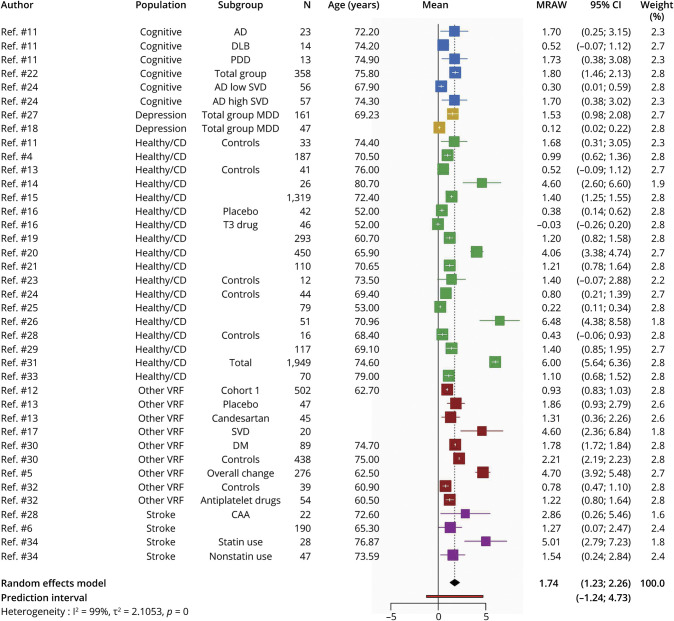
Random-Effects Meta-analysis of Raw Mean WMH Volume Change in Milliliters Over Median of 2 Years, Range 0.25–8.1 Years Squares represent means and bars the 95% CI. AD = Alzheimer disease; CAA = cerebral amyloid angiopathy; DLB = Lewy body dementia; DM = diabetes mellitus; healthy/CD = healthy/community-dwelling; MDD = major depressive disorder; MRAW = raw means; PDD = Parkinson disease dementia; SVD = small vessel disease; VRF = vascular risk factor; WMH = white matter hyperintensity.

We identified 27 articles^4-6,11-34^ (total 7,411 participants) that reported raw WMH volume change over time. The overall time between scans was on average 2.7 years (SD = 1.65; median = 2 years; range 0.25–8.1 years). We combined all data from all populations in 1 meta-analysis (Figure 2). Overall WMH increased by mean 1.74 mL over time (95% CI 1.23–2.26 mL with a PI of −1.24 to 4.73 mL).

#### Change in %ICV

**Figure 3:**
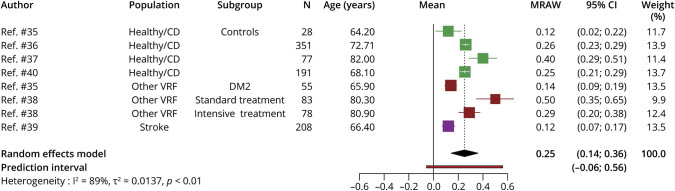
Random-Effects Meta-analysis of Mean WMH Volume Change as %ICV Over Median of 3 Years, Range 1.9–6.7 Years Squares represent means and bars the 95% CI. DM2 = diabetes mellitus type 2; healthy/CD = healthy/community-dwelling; MRAW = raw means; VRF = vascular risk factorWMH = white matter hyperintensity.

Six studies reported WMH volume and their volume change over time as %ICV.^35-40^ (total 1,071 participants), with time between MRI mean 3.5 years (SD = 1.54, median = 3; range 1.9–6.7) were meta-analyzed (Figure 3). WMH volume as %ICV shows an increase of 0.25 (95% CI 0.14–0.36; PI −0.06 to 0.56) %ICV.

#### WMH Change in Milliliters per Year

**Figure 4:**
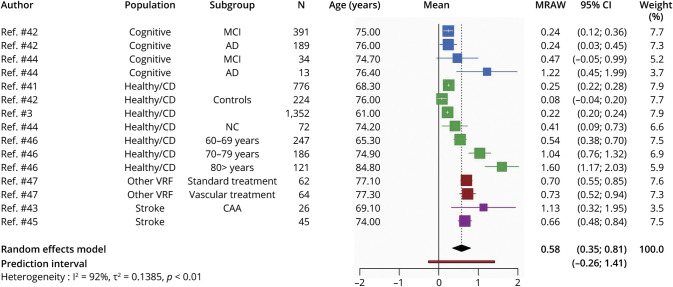
Random-Effects Meta-analysis of Mean WMH Volume in Milliliters Per Year Squares represent means and bars the 95% CI. AD = Alzheimer disease; CAA = cerebral amyloid angiopathy; healthy/CD = healthy/community-dwelling; MCI = mild cognitive impairment; MRAW = raw means; NC = normal control; VRF = vascular risk factor; WMH = white matter hyperintensity.

Eight studies^41-47,e6^ (total 3,802 participants) reported the unadjusted mean WMH change per year (Figure 4). Overall, mean WMH change showed an increase of 0.58 (95% CI 0.35–0.81; PI −0.26 to 1.41) mL/y.

### Subgroup Analyses

For explorative purposes, we examined WMH change per population type and, when possible, by age and time lapse between scans. As suggested by reviewers, we examined WMH change in milliliters and baseline WMH volume in milliliters (eAppendix 3 and eFigure 1, links.lww.com/WNL/C292).

### Population

We performed meta-analyses per population group for all 3 ways of reporting WMH change (milliliters, %ICV, and milliliters per year). Where studies reported treatment groups, control or case groups, these subgroups are reported. Not all population groups were available for %ICV and milliliters per year.

WMH raw volume increased by 1.78 mL (95% CI 0.83–2.73; PI −2.18 to 5.75; eFigure 2, links.lww.com/WNL/C292) in the healthy and community-dwelling population over 2.9 ± 1.3 years (range 1–5.3 years).^4,11,13-16,19-21,23-26,28,29,31,33^ WMH %ICV increased by 0.26 %ICV (95% CI 0.09–0.43; PI −0.20 to 0.71; eFigure 3) over 1.9–6.7 years^35-37,40^ and the annual rate by 0.56 mL/y (95% CI 0.06–1.06; PI −0.86 to 1.98; eFigure 4).^41,42,44,46,e6^

In people with memory complaints^22^ and dementia,^11,24^ WMH increased by mean 1.17 mL (95% CI 0.40–1.94; PI −0.87 to 3.20; eFigure 5, links.lww.com/WNL/C292). Among mild cognitive impairment and Alzheimer disease (AD) groups, WMH increased by 0.27 mL/y (95% CI 0.02–0.51; PI −0.07 to 0.60; eFigure 6).^42,44^

In people with depression, WMH volume increased by 1.19 mL (95% CI −1.81–4.18; PI −6.90 to 9.27; eFigure 7, links.lww.com/WNL/C292).^18,27^ Within the depression populations, a group with incident dementia at follow-up^27^ presented the largest mean WMH volume increase with 4.52 mL (95% CI 2.25–6.79), whereas the raw mean volume changes for the other depression groups range from 0.08 to 1.10 mL.

Among populations recruited because of VRFs, that is, participants with diabetes,^12,35^ SVD,^5,17^ multiple risk factors,^32,47^ hypertension,^13,38^ and vascular disease or high risk of vascular disease,^30^ WMH volume also increased on average, including increases of 2.02 mL (95% CI 0.95–3.09; PI −1.19 to 5.23; eFigure 8, links.lww.com/WNL/C292),^5,12,13,17,30,32^ 0.30 %ICV (95% CI −0.14–0.74; PI −2.22 to 2.81; eFigure 9),^35,38^ and 0.71 mL/y (95% CI 0.53–0.89; PI not calculable; eFigure 10).^47^ Data on patients with stroke were available for WMH volume in milliliters, increase of 2.46 mL (95% CI −0.21–5.12; PI −4.50 to 9.41; eFigure 11, links.lww.com/WNL/C292),^6,28,34^ and milliliters per year, an increase of 0.72 mL/y (95% CI −1.32 to 2.76; PI not calculable; eFigure 12).^43,45^

### Time Between Scans

**Figure 5:**
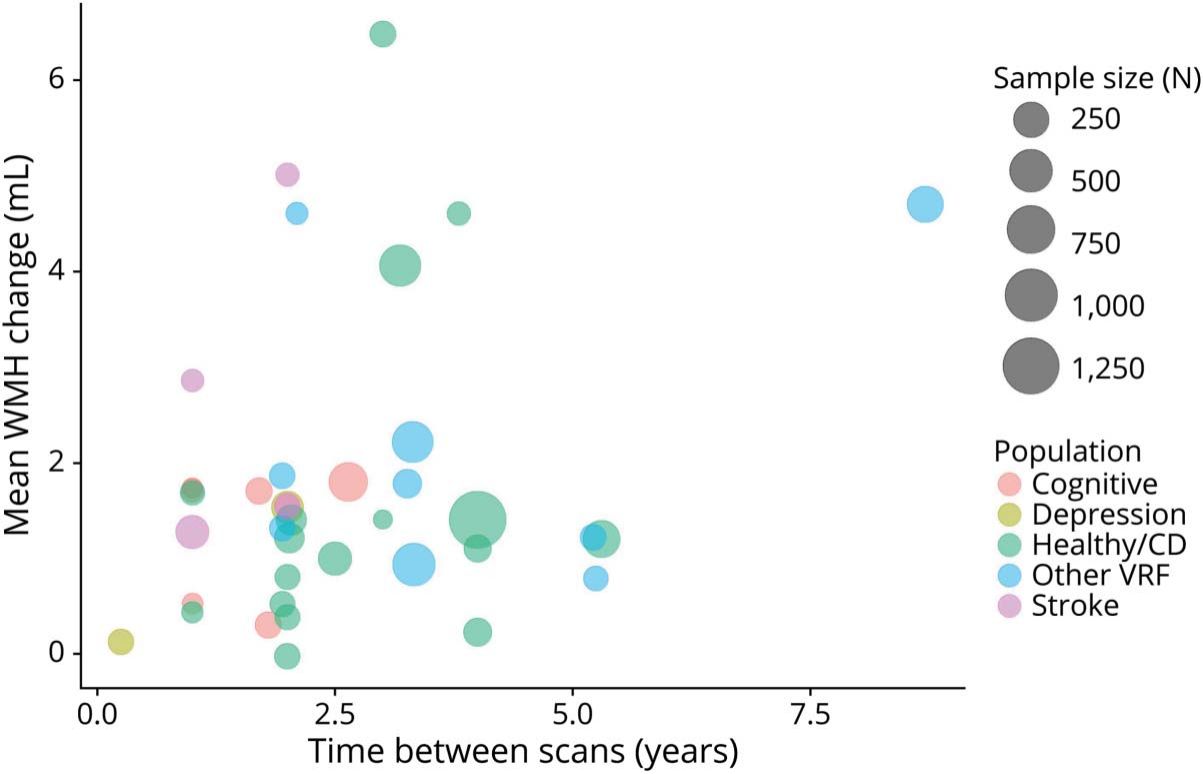
Bubble Plot of Mean WMH Change (Milliliters), in Individual Studies, Related to Time Between Scans (Years) Points in the plot are scaled by sample size, and the color of points refers to the population group. Healthy/CD = healthy/community-dwelling; VRF = vascular risk factor; WMH = white matter hyperintensity.

**Figure 6:**
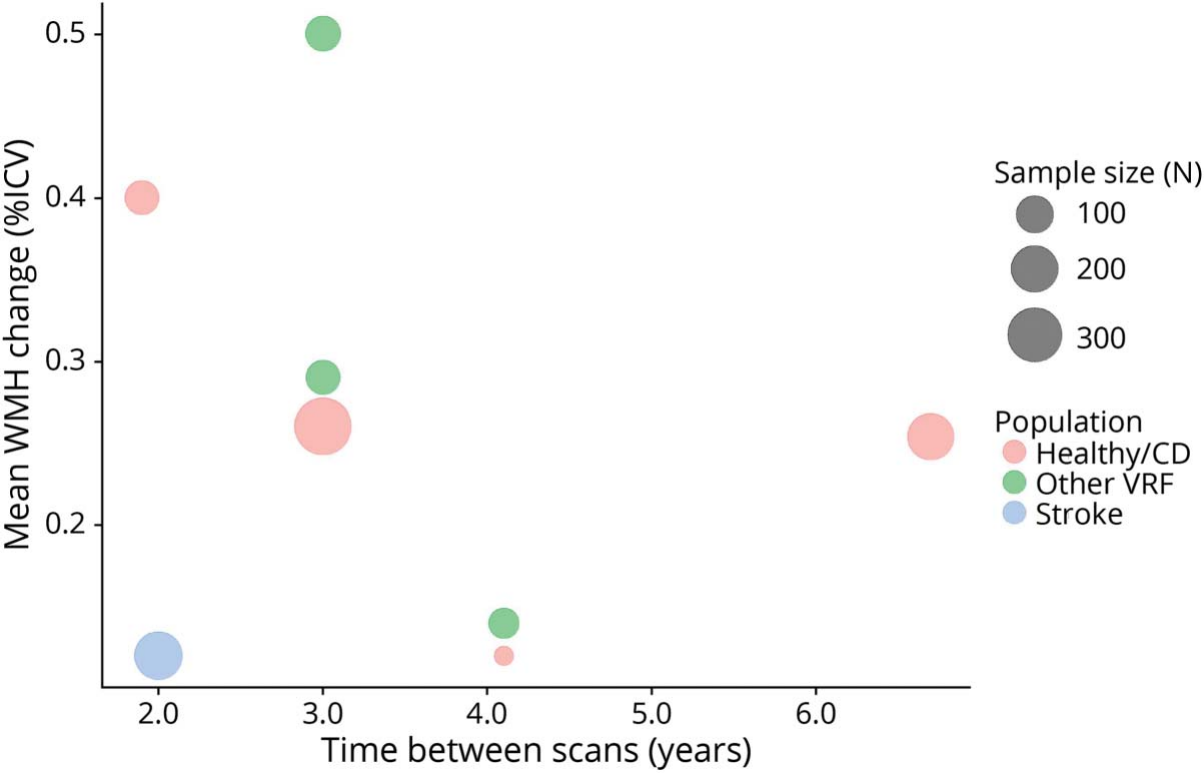
Bubble Plot of Mean WMH Change (%ICV), in Individual Studies, Related to Time Between Scans (Years) Points in plot are scaled by sample size, and the color of points refers to the population group. %ICV = percentage of intracranial volume; healthy/CD = healthy/community-dwelling; VRF = vascular risk factor; WMH = white matter hyperintensity.

Most studies had a follow-up time between scans of around 2 years. The 27 articles reporting raw volume in milliliters had a follow-up time of 2.7 ± 1.65 years (median 2, range 0.25–8.7 years). Longer follow-up times between scans appeared associated with larger WMH increase in milliliters (Figure 5). In a similar bubble plot of mean WMH change as %ICV (3.5 ± 1.54 years, median 3; range 1.9–6.7), there is no clear relation between longer time between scans and larger WMH change (Figure 6), but there were far fewer studies.

### Age

**Figure 7:**
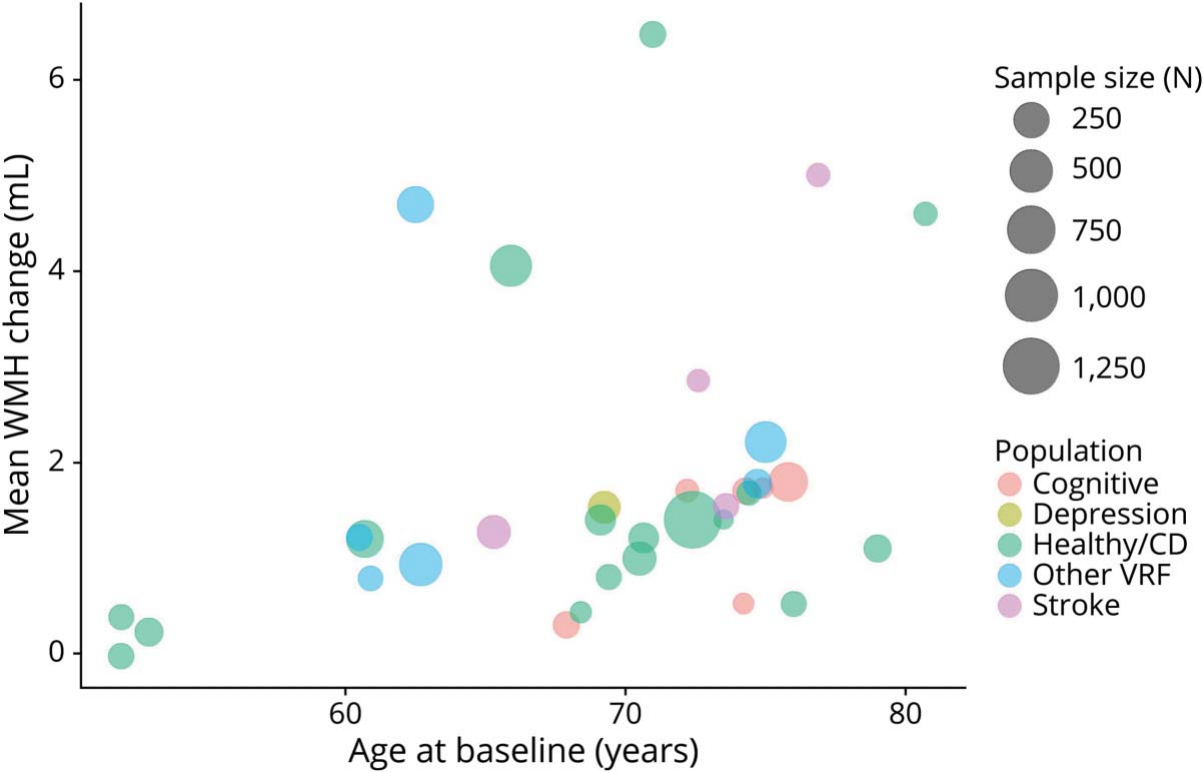
Bubble Plot of Mean WMH Change (Milliliters) Related to Age at Baseline (Years) Points in plot are scaled by sample size, and the color of points refers to the population group. Healthy/CD = healthy/community-dwelling; VRF = vascular risk factor; WMH = white matter hyperintensity.

Patterns in the bubble plots of WMH change vs mean age at baseline suggest that WMH volume change increases at older ages across WMH in milliliters (Figure 7), %ICV (eFigure 13, links.lww.com/WNL/C292), and milliliters per year (eFigure 14), with a younger mean age at baseline generally corresponding to smaller WMH change over time.

### WMH Regression

Thirteen of the 41 articles (eTables 3–5, links.lww.com/WNL/C292) included in the meta-analysis mentioned WMH volume regression, of which WMH volume regression was discussed by 8/13 articles,^4-6,16,24,26,45,46^ but only 7/8 articles^6,11,16,22,23,25,28^ provided data. One article^46^ did not mention regression but showed WMH regression in a figure with individual trajectories.

WMH regression was found in healthy/community-dwelling participants (∼34%^4^ and 17%^26^) and participants with stroke (4%^45^ and 37%^6^). Areas of WMH shrinkage were found in participants with AD with high SVD load, with less WMH regression in cognitively normal controls who had less WMH at baseline than the patients with AD and high SVD load.^24^ Over 9 years of follow-up^5^ (n = 276), 1 participant (0.4%) showed net WMH regression, whereas 9% showed regression in the first follow-up period (2006–2011) and 2% in the second period (2011–2015). Factors associated with WMH regression were not found.^48^ Observations of WMH regression from articles not included in meta-analysis (eTable 6, links.lww.com/WNL/C292) can be found in eAppendix 2 and associations with WMH change in eAppendix 4.

## Discussion

Our meta-analyses suggest that although WMH volumes increase on average, WMH volumes also regress, with WMH volume regression occurring explicitly in up to one-third of participants. The PIs of the main analyses of WMH change capture the extent of WMH volume regression (−1.24 mL; −0.06 %ICV; −0.25 mL/y) and increase (4.73 mL; 0.56 %ICV; 1.46 mL/y). We show that WMH regression can occur in all typical populations affected by SVD, greater WMH volume change might occur at older age, and WMH regression might occur over a wide range of follow-up times.

As WMH progression is the main focus of most articles and WMH regression is regarded as an accidental finding or even an error, the underlying mechanisms of WMH regression are unknown, or whether regression represents improvement in tissue health and translates to a positive effect on clinical outcomes.^48^ However, some evidence suggests that regression is linked to less cognitive decline, recurrent stroke, or dependency.^49^ Furthermore, if patients with less WMH progression have less cognitive impairment than those with more WMH progression, there is reason to think that WMH regression might translate to even better clinical outcomes, for example, cognition, motor deficits, or dependency.

The articles included had several limitations. First, methodologies to measure and report WMH volume change, that is, milliliters, %ICV, or milliliters per year, varied and complicated the comparison of volume change, and means that the findings should be interpreted with caution. Although there are many methods to assess WMH volume, including artificial intelligence approaches, there is little cross-validation or standardization. Furthermore, methods specifically addressing WMH volume change that account for registration steps are only beginning to emerge.^50^ Second, articles that report WMH volume change as an annualized rate might represent a bias by assuming that change is linear. We did not find a clear relation between longer follow-up times and larger WMH volume change, but this may reflect the heterogeneity of studies, populations, and follow-up times. A study with a 9-year follow-up period^5^ shows that WMH volumes can both increase and decrease within the 9 years and that more people had WMH regression in the first 5 years than in the second part. Thus, studies reporting annualized rates should also report the absolute change over time at the final time point. Our analyses were limited by not being able to examine factors related to regression since too few articles examined these. For the same reason, we were unable to assess the effect of interventions on WMH volume regression.

During the screening phase of the systematic review, 10% of the titles and abstracts and 20% of the full texts were screened by 2 reviewers. This might have led to missing some relevant articles. However, the agreement between reviewers was good, and any disagreements were discussed with a third reviewer. The strengths of our review include a comprehensive literature search of WMH volume change using different measurements of WMH volume; subgroup exploratory analyses into differences between populations, age and follow-up times; and a good geographical coverage of included studies. The review uses PIs to show least and most change, rather than only CIs, which focus on the mean and thus obscure the true limits of change. Hence, the review demonstrates the range of interindividual differences in WMH progression volume that may have been overlooked through the tendency in previous studies to focus solely on WMH progression.

Future studies should examine WMH change including the possibility of WMH regression and investigate WMH change over a long period. The median follow-up times in our main analyses were 2 years (milliliters) and 3 years (%ICV), providing little evidence over longer follow-times.^2^ Longer follow-up times and scans at multiple time points would provide more information on trajectories of volume change and dynamics of WMH. It would be very valuable to look into any anatomic patterns of WMH change, for example, locations of stable WMH, regression, or progression. WMH changes might have different underlying mechanisms in subtypes of SVD, for example, in cerebral amyloid angiopathy, which is currently underrepresented and should be assessed. Also, WMH related to other causes than SVD, or comorbidities, might get mistaken for SVD. More detailed studies are needed to work out the underlying mechanisms as that cannot be concluded from the current analyses. In addition, examination of possible pathologic, imaging, and clinical factors related to WMH regression is vital as the exact underlying mechanisms and clinical consequences are unknown. Finally, we encourage studies to sufficiently report WMH analysis methods and scanning details, including any changes or interference.

In conclusion, our results indicate that WMH volumes can regress over time in diverse populations, whereas net WMH volume might progress. However, little is known about underlying mechanisms of WMH volume regression, which might represent an opportunity to prevent WMH or develop new interventions and delay the progression of WMH and its devastating clinical consequences.

## Glossary

%ICV: percentage of intracranial volume
AD: Alzheimer disease
IQR: interquartile range
PI: prediction interval
SVD: small vessel disease
VRF: vascular risk factor
WMH: white matter hyperintensity
